# A steep decline of malaria morbidity and mortality trends in Eritrea between 2000 and 2004: the effect of combination of control methods

**DOI:** 10.1186/1475-2875-5-33

**Published:** 2006-04-24

**Authors:** Peter M Nyarango, Tewolde Gebremeskel, Goitom Mebrahtu, Jacob Mufunda, Usman Abdulmumini, Andom Ogbamariam, Andrew Kosia, Andemariam Gebremichael, Disanayike Gunawardena, Yohannes Ghebrat, Yahannes Okbaldet

**Affiliations:** 1 Orotta School of Medicine, P.O. Box 10549, Asmara, Eritrea; 2 National Malaria Control Programme, Ministry of Health, Eritrea; 3 Division of Disease Prevention and Control, Ministry of Health, Eritrea; 4 Department of Research & Human Resource Development, Ministry of Health, Eritrea; 5 WHO Eritrea Country Office, Asmara, Eritrea; 6 Research Triangle Institute, North Carolina, USA

## Abstract

**Background:**

Malaria is a huge public health problem in Africa that is responsible for more than one million deaths annually. In line with the Roll Back Malaria initiative and the Abuja Declaration, Eritrea and other African countries have intensified their fight against malaria. This study examines the impact of Eritrea's Roll Back Malaria Programme: 2000–2004 and the effects and possible interactions between the public health interventions in use.

**Methods:**

This study employed cross-sectional survey to collect data from households, community and health facilities on coverage and usage of Insecticide-Treated Nets (ITNs), Indoor Residual Spraying (IRS), larvicidal activities and malaria case management. Comparative data was obtained from a similar survey carried out in 2001. Data from the Health Management Information System (HMIS) and reports of the annual assessments by the National Malaria Control Programme was used to assess impact. Time series model (ARIMA) was used to assess association.

**Results:**

In the period 2000–2004, approximately 874,000 ITNs were distributed and 13,109 health workers and community health agents were trained on malaria case management. In 2004, approximately 81% households owned at least one net, of which 73% were ITNs and 58.6% of children 0–5 years slept under a net. The proportion of malaria cases managed by community health agents rose from 50% in 1999 to 78% in 2004. IRS coverage increased with the combined amount of DDT and Malathion used rising from 6,444 kg, in 2000 to 43,491 kg, in 2004, increasing the population protected from 117,017 to 259,420. Drug resistance necessitated regimen change to chloroquine plus sulfadoxine-pyrimethamine. During the period, there was a steep decline in malaria morbidity and case fatality by 84% and 40% respectively. Malaria morbidity was strongly correlated to the numbers of ITNs distributed (β = -0.125, p < 0.005) and the amount (kg) of DDT and Malathion used for IRS (β = -2.352, p < 0.05). The correlation between malaria case fatality and ITNs, IRS, population protected and annual rainfall was not statistically significant.

**Conclusion:**

Eritrea has within 5 years attained key Roll Back Malaria targets. ITNs and IRS contributed most to reducing malaria morbidity.

## Background

The success in malaria eradication achieved in Europe and North America during the 19^th ^and 20^th ^centuries has not been replicated anywhere in Sub-Saharan Africa and most tropical countries. This is despite better scientific understanding of the biology of the vector, treatment methods and other means of malaria prevention and control. Malaria causes one million deaths annually in Africa especially among vulnerable groups of pregnant women and children under five years of age [1]. This can be attributed to a number of factors including inadequate preventive measures for the groups at the highest risk of contracting malaria such as pregnant women and children under five years of age and drug resistance [2].

While, malaria eradication has eluded most tropical countries the use of conventional information sources often underestimates the true incidence [3]. Resurgences of severe malaria and in recent years, recurrent epidemics invariably involving falciparum malaria have been reported from many tropical countries [4].

Overtime, there have been several global initiatives to control malaria. The Roll Back Malaria [5] and the Abuja Declaration [6] are the recent attempts to coordinate efforts and provide more resources to reduce the malaria burden in the world. Mostly, the strategies used aimed at primary prevention through vector control or use of personal preventive methods such as bed nets, mosquito repellants, chemoprophylaxis and finally, through effective case management and medication.

In many developing countries there is variable success in vector control using ITNs, IRS with DDT and other agents [7]. The factors which influence the effectiveness of malaria prevention and control include national policies, community and personal prevention, community awareness, quality of health care, facility and health personnel competence as well as effective monitoring of anti-malarial drug resistance and timely change of drug regimen when resistance occurs [8].

In Eritrea, 67% of the population live in malaria endemic areas. Of this population 18% are children aged five years and below and 22% are women aged between 15 and 45 years. Falciparum malaria is predominant in Eritrea and is mainly transmitted by *Anopheles arabiensis *[9], which is known to be endophilic. Eritrea is inhabited by more than 13 different species of anopheline mosquitoes all capable of spreading the disease and with varying geophysical habitats [10]. Also, inoculation rates have a high seasonal variability, with peak inoculation rates during the rainy season and minimal or no transmission during the dry season [11].

Malaria is known to negatively impact on socio-economic development of Eritrea. About 7 to 12 days are lost per episode of malaria, thus having an enormous impact on the productive labour force [12]. The average cost for treating an episode of uncomplicated malaria is about 2.00 USD and about 7.00 USD for severe cases [12]. These treatment costs are significant for a country with a per capita GDP below 200 USD. Also, in 1999, malaria accounted for 31.5% of the total outpatient morbidity and 28.4% of all admissions. Malaria was responsible for 19.6% of hospital admissions among children under five years of age.

In view of the public health importance of the burden of malaria in Eritrea, in 1999, the Ministry of Health organized a national workshop on Roll Back Malaria to develop control strategies and to launch a 5-year Malaria Control Programme. The outcome of the workshop was a national resolve to control malaria as contained in the Mandefera Declaration and the plan of action for the period 2000–2004. The core objectives of the plan were to reduce malaria morbidity and mortality by 80% from the 1999 levels [13]. During the period 1995–1998, malaria control activities had succeeded in developing policies and guidelines, and the training of health professionals on malaria control and treatment resulting into 1200 active community agents, distribution of 81,036 ITNs and 76,209 houses were subjected to IRS [13].

Implementation of the plan of action commenced immediately at the beginning of 2000 with financing from the Eritrean Government, World Bank, WHO, UNICEF, USAID and later Global Funds. During the same year, the country adopted the Abuja Declaration targets and goals for the purposes of programme management. The Abuja targets aimed at reducing malaria burden by at least 60%, and ensuring that at least 60% persons suffering from malaria had access to prompt treatment using anti-malarial drugs. Further, at least 60% of persons at risk of malaria, particularly children under five and pregnant women will benefit from a suitable combination of personal and community protective measures such as insecticide treated nets (ITNs). Finally, the Abuja declaration requires that at least 60% of pregnant women who are at risk of malaria would have access to intermittent preventive treatment through use of SP [6].

This study is aimed at assessing the 5-year achievements of the Roll Back Malaria Programme in Eritrea, 2000–2004. The specific objectives of this study were to assess trends in malaria morbidity and mortality rates in the country, and the effectiveness of the various public health measures used in Eritrea's Malaria Control Programme. The assessment indicators were derived from Roll Back Malaria Initiative, the Abuja Declaration and Eritrea's Mandefera Declaration [12-14].

## Methodology

### Study population

Eritrea is geographically divided into six regions or *zobas*. There are four lowland zobas, two of them coastal and to the east of the country (altitude: 0–1,000 m) and the other two in the west of the country (altitude: 600–1,000 m). The eastern and western lowlands stride the remaining two highland *zobas *that are centrally located in the country (altitude: 1,500–2,000 m). The country is stratified on the basis of malaria risk based on the underlying geo-physical stratification and seasonality, as well as yearly probability of cases [9] (incidence). Four *zobas *(Anseba, Debub, Gash Barka and Northern Red Sea) meet the classification for high malaria risk regions, representing 67% of the population and almost four-fifths of the landmass.

### Programmeme strategies for NMCP 1999–2004

The foci of the Eritrea malaria control plan were: primary prevention through selective vector control and behavioural practices, mortality and morbidity reduction through effective case management and epidemic control, health systems strengthening, and implementation of an effective information and communication strategy. Primary prevention focused on vector control and personal behaviour change specifically through the consistent use of bed nets. Vector control was done at two levels of source reduction through larval control and through adult mosquito control using indoor residual spraying (IRS). The changes in behavioural practices to prevent mosquito bites and to control adult mosquitoes consisted of distribution, re-treatment and use of ITNs. For the high-risk *zobas*, the national policy applies equity weightings so as to bias resource allocation in the favour of these *zobas*. For example one free ITN is to be issued to every pregnant woman or child who is resident in any of the high-risk zobas. This is to target interventions and to guarantee access of an essential preventive tool to the most vulnerable groups who, while being at risk of infection, would otherwise not afford this intervention.

To strengthen effective case management, training of health personnel for community and health facility care, including technicians, continued throughout the plan period. Integrated Management of Childhood Illnesses (IMCI) was subsequently adopted as a means of addressing malaria for children [15].

Malaria surveillance monitored four factors of monthly rainfall, the weekly number of new malaria cases in relation to the set epidemic thresholds, and resistance to insecticides commonly used for spraying or treatment of nets as well as resistance to chloroquine and the newly introduced first line combination treatment.

### Case definition

Malaria is diagnosed using direct microscopy in hospitals or health centres and by use of Rapid Diagnostic Test Kit in lower level facilities. Clinical diagnosis is the method used for case definition by community agents, drug vendors and community members.

### Study design

The study design was composed of a retrospective component and a cross-sectional survey. In the retrospective component, data was obtained from Health Management Information System (HMIS) of the Ministry of Health, quarterly and annual reports of the National Malaria Control Programme (NMCP) and reports of the annual assessment workshops by NMCP. The Ministry collects HMIS data from all health facilities in the country, and annually the HMIS data achieved over 90% completeness for the period 2000–2004 [16]. In addition, data was obtained from published reports of the midterm evaluation of the Roll Back Malaria programme 1999–2004 that was carried out in 2001. The mid-term survey and the end of programme survey used the same data collecting instruments and study design. Rainfall data was obtained from the Ministry of Agriculture, Eritrea. The monthly rainfall data was collected from 22 meteorological stations located in all the six zobas of the country. Lastly, interviews on programme design and implementation process were carried out and information obtained from the NMCP offices and *zoba *malaria coordinators.

The study was a three-part one composed of desk review, health facility and community survey. The health facility survey generated data on training, availability of equipment, drugs and other supplies essential for diagnosis and treatment of malaria as well as quality of care. Data for the cross sectional study was obtained during the final evaluation of the 5-year malaria control programme conducted during the period September-December 2004, which coincided with the peak malaria transmission period.

The desk review component assessed programme management and impact of interventions using data from the HMIS and the NMCP. In the community survey the information gathered included ownership and use of ITNs, health seeking behaviour and community participation in IRS and ecological interventions.

The methodology for the health facility and community survey is described below.

### Sampling

#### Health facility survey

The health facility survey was conducted in 15% of health facilities in all the four malaria endemic zobas of the country (Gash-Barka, Debub, Anseba and Nothern Red Sea). A total of 28 facilities were randomly selected and included in the facility survey. Considering the seasonal nature of malaria, the survey was conducted over a period of two weeks during the months of October and November, which is the peak malaria transmission season in the four *zobas*. The target population was the general population with a bias towards children under-five years of age, pregnant women and children over five who report to health facilities with fever/malaria during the survey period. All children under five years of age coming to the health facility with fever, during the survey period were included in the sample. The sample frame was made up of a list of all health facilities obtained from the HMIS. Facilities were selected by type, i.e hospitals, health centers and health stations within each of the 4 *zobas*. At least one hospital in each *zoba *was included in the sample.

The object of the health survey was to assess the quality of care in the health facilities of the endemic zones through direct observation and exit interview. All individuals who presented to the selected health facilities with malaria or fever during the two-week period of the survey were to be observed at the stage of consultation and interviewed at exit time. During the observational stage trained research assistants, using a checklist, recorded the practice of clinical skills: whether clinicians asked caregivers or patients about fever, took temperature measurements, requested for blood tests for malaria, the drugs that were prescribed and if patients or caregivers received counseling.

#### Community survey

The community component of the survey was designed to cover the population residing in the same four high malaria endemicity *zobas*. The sample size calculation was based on input from the National Statistics and Evaluation Office (NSEO) that aimed to generate reliable estimates on malaria indicators for the different segments of the population, (children under 5 years of age, persons aged 5 years and above, and pregnant women). The proportion of pregnant women who slept under the mosquito bed net on the previous night (7.6%) rounded to 8.0% (p = 0.08), based on the 2002 Eritrea Demographic and Health Survey (EDHS)[17], was used as the starting point of the calculations, with an assumed relative error of 20% (C.V = 0.20).

**n = q/((C.V)**^2^*p); where q = 1-p

n = 0.92/(0.2*0.2*0.08) = 288

This implies that the survey required a sample size of 288 pregnant women for the four zobas combined together. In the EDHS 2002, a household had on an average of about 0.2 pregnant women. This made the number of households to be selected in this sample to be about 1440 (i.e., 288/0.2).

Moreover, a response rate of 95% was obtained in the 2002 EDHS for the household survey. Adjustment for this response rate made the sample of households to be covered about 1,516 (1440/0.95) and rounded to 1,520 households. The total of 1,520 households (HHs) was allocated equally among the 4 *zobas *to get equal precision for each *zoba*.

The sample design adopted for the survey was a two stage stratified cluster design. At the first stage, clusters (rural/urban) were selected as Primary Sampling Units (PSU) and at the second stage households were selected as Secondary Sampling Units (SSU). The domain of the study was all the four zobas combined together.

In each *zoba *a total of 38 (i.e. 380/10) clusters (villages or urban areas) were selected. The survey aimed to reach 10 households in each cluster. The frame used for this purpose was the list of villages and urban clusters prepared by the respective *zoba *administration offices in 2004.

### Statistical analysis

Malaria incidence rates and case fatality rates in the country were computed covering the period 2000–2004. The Time Series analysis was used to test for any association between morbidity and/or mortality being dependent variables and the intervention measures as independent variables. The aggregated data from all six *zobas *of the country was used in testing for the impact of the various public health interventions.

### Limitations

The data set on interventions, malaria morbidity and mortality covers only a five-year period, which is too short to generalise on long-term trends and provide adequate statistical power in all instances of regression analysis and for each of the variables of interest. However, on behaviour change, these shortcomings are corrected for through comparative analysis of cross-sectional data from the same study population and using similar tools, collected at two intervals in 2001 and 2004. The sample size calculation for the community and facility surveys was designed realise validity at the national level and not the zoba level.

## Results

### Sample size coverage

All the selected health facilities were reached and a total of 231 patients or caretakers were interviewed. Direct observations during clinical management at the health facility were made on the same number of patients. Out of the estimated sample of 288 pregnant women, 238 (82.6%) were identified during the community survey (Table 1).

**Table 1:** Expected sample sizes and samples size achieved per *zoba*

**Zone (Zoba)**	**Number of households**	**Sick children under five years**	**Pregnant women**	
	
	**Minimum sample size expected**	**Sample size reached**	**Minimum sample size expected**	**Sample size reached**
Anseba	380	71	72	64
Debub	380	48	72	61
Gash-Barka	380	42	72	55
Northern Red Sea	380	35	72	58

**TOTAL**	**1520**	**196**	**288**	**238**

### Findings from review of HMIS data

**Figure 1:**
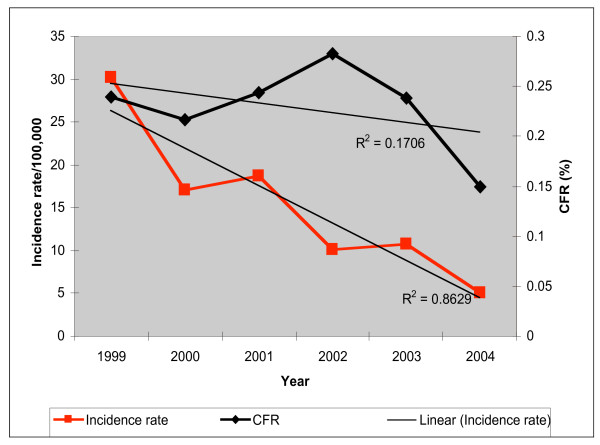
Malaria morbidity and case fatality rates 1999–2004.

Analysis of data from the HMIS, revealed that during the period 2000–2004, the incidence rate of malaria and case fatality rate in Eritrea declined precipitously. The overall outpatient malaria incidence rate dropped by 83.33% while the malaria case fatality rate decreased from 0.21% to 0.14% (Figure 1). An increase in case fatality rate trends during the period 2000–2002 was subsequently followed by marked decline to below the 1999 levels.

The reduction in morbidity and case fatality rate varied across the different zobas with the greatest reduction occurring in Anseba (Table 2).

**Table 2:** Trends in malaria incidence rate (IR) and case fatality rate (CFR) by zoba

Year	Anseba		Debub		G. Barka		Maekel		Northern Red Sea		Southern Red Sea	
IR	CFR	IR	CFR	IR	CFR	IR	CFR	IR	CFR	IR	CFR	

1999	1938.7	0.3	2198.0	0.2	7696.5	0.2	461.6	0.1	2270.5	0.3	940.7	1.3
2000	830.1	0.4	1223.2	0.2	4462.1	0.2	190.8	0.4	1717.9	0.2	1059.8	0.9
2001	686.4	0.7	1196.2	0.2	4636.6	0.2	360.2	0.2	2523.4	0.2	1082.2	0.4
2002	327.9	0.4	754.3	0.2	2546.8	0.4	272.4	0.1	1053.8	0.1	1018.5	0.1
2003	332.7	0.7	1031.8	0.3	2691.3	0.2	253.7	0.3	820.9	0.1	825.6	0.2
2004	57.0	0	503.2	0.1	1318.0	0.1	124.5	0.3	328.1	0.4	490.0	0

According to the HMIS data, the number of breeding sites in the country eliminated annually by filling water pools increased by 70% (Table 3). Similarly, countrywide larvicidal control efforts were carried out with the proportion of sites treated annually rising more than fivefold. The number of houses covered through IRS trebled with the estimated number of people protected using this method rising to twice the number in 1999. Studies on vector resistance to DDT and other commonly used insecticides revealed high efficacy throughout the period. No resistance was reported.

**Table 3:** Malaria control activities in all *zobas *of Eritrea, 2000–2004

	**2000**	**2001**	**2002**	**2003**	**2004**
Number of houses sprayed	39,838	76,754	60,433	97,069	92,107
Malathion used (kg)	2,399	7,904	5,555	21,890	30,388
DDT used (kg)	4,045	8,362	8,500	17,423	13,103
Population protected by IRS	117,017	202,652	159,551	227,675	259,420
Breeding sites filled (pools)	15,988	23,810	25,355	22615	27494
Breeding sites treated (pools)	11,691	7,690	12,547	67,684	33,442
Abate (Temephos) used (litres)	14.9	18.5	145.0	90.5	80.2
Population participating in treating and filling breeding site	54,219	72,824	51,666	48,948	111,494
ITNs distributed	127,863	67,708	276,038	187,709	214,752

**Figure 2:**
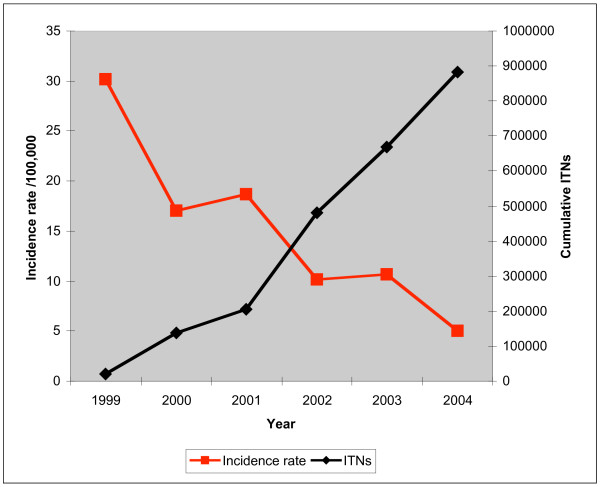
Distribution of ITNs and trends in malaria incidence rate.

During the period 2000–2004, approximately 874,070 free ITNs were distributed to pregnant women and children in the four high malaria transmission regions. Results from the community survey data revealed that 23.7% of the ITNs in the community were obtained through out of pocket household purchase. The rate of the annual increase in the distribution of ITNs is strongly correlated to the declining trend in malaria morbidity. The largest decrease in the incidence rate of malaria was during the year 1999. Thereafter, the decrease in malaria morbidity was a mirror image of the annual distribution of ITNs (Figure 2).

During the period 2000–2004, there was a steady increase in the total number of personnel trained annually (Table 4). The role of community health agents played in case management gained prominence during the study period. The proportion of cases managed by CHAs rose from approximately 50% in 1999 to 78% in 2004.

**Table 4:** Number of personnel trained in case management

Year	Community Health Agents	Health workers	Laboratory Technicians	Rural drug Vendors	Military health personnel	Community members
2000	936	370	15	0	593	1121
2001	1419	497	0	0	314	673
2002	1077	274	15	66	0	666
2003	1382	160	41	0	0	1176
2004	1446	80	62	37	0	689

**Total**	**6260**	**1381**	**133**	**103**	**907**	**4325**

**Figure 3:**
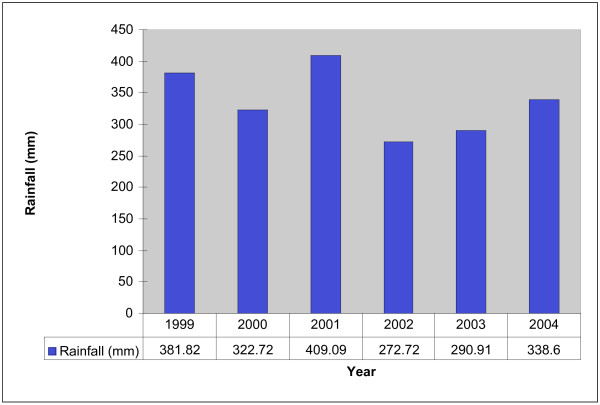
Average annual rainfall (mm) in Eritrea 1999–2004.

Between 1992 and 2004, there was increase in rainfall until 1997. This was followed by a decline in the annual rainfall dropping during the period 2000–2004 to a pattern similar to the one of 1992–1995 (Figure 3). No epidemics were detected during the study period. The last reported epidemic was in 1998.

In 2002, resistance to chloroquine as the first line single drug-treatment regimen reached 6% necessitating introduction of combination first line therapy using chloroquine and sulfadoxine-pyrimethamine. In 2004 resistance to this combination of therapy rose to 4%, which was less than the threshold for changing this combination therapy. There was no resistance detected to DDT, Malathion or any of the insecticides used for the control of adult mosquitoes.

### Findings from community surveys

**Figure 4:**
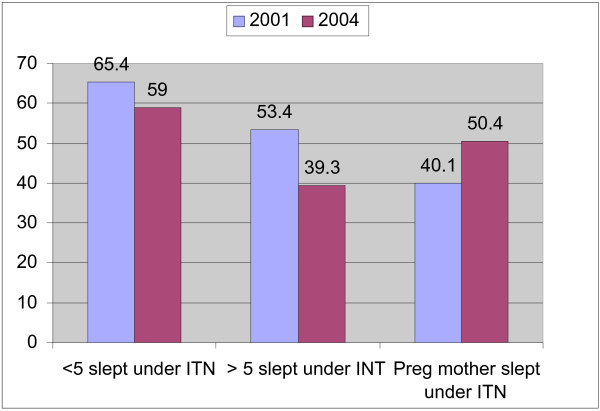
Proportion of children, adults and pregnant women sleeping under ITN.

In 2001, 80% of surveyed households owned at least one net of which 67.9% were ITNs and 87.7% of the ITNs had been re-treated. The corresponding proportions of ITNs in 2004 were 79%, 73% and 62% respectively revealing a significant reduction in the proportion of recently treated nets i.e. within 6 months of the survey. The use of nets was slightly lower than ownership. Although the overall use of ITNs was relatively high, utilization had slightly declined in 2004 compared to the practice in 2001(Figure 4). The ownership and use of ITNs varied by zoba with Anseba having the highest coverage and utilization rates (Table 5)

**Table 5:** Availability of ITNs in households by *zoba*

		**% Households own ITN**		**% ITN Re-treatment**	
		
**Zoba**	**N =**	**>1 ITN declared**	**>1 ITN seen**	**>1 ITN seen**	**> 1 ITN in last 6 months**
Anseba	386	98%	97%	96%	94%
Debub	380	84%	82%	75%	69%
Gash-Barka	377	77%	75%	68%	67%
Northern Red Sea	380	64%	62%	52%	18%

**TOTAL**	**1523**	**81%**	**79%**	**73%**	**62%**

There was wide geographical variation in the usage of ITNs with Anseba recording the highest rates of use (Table 6). Children under 5 years of age attained higher usage of ITNs than that amongst older persons.

**Table 6:** Proportion of persons sleeping under Net/ITN in previous night by *zoba *and age

	**Age < 5 years**			**Age > 5 years**			**All ages**		
**Zoba**	**N =**	**Any Net**	**ITN**	**N =**	**Any Net**	**ITN**	**N =**	**Any Net**	**ITN**

Anseba	**317**	79.8%	77.6%	**1713**	72.3%	71.0%	**2035**	73.6%	72.1%
Debub	**304**	56.3%	51.6%	**1654**	40.3%	35.2%	**1965**	42.8%	37.8%
Gash-Barka	**271**	57.2%	51.3%	**1653**	43.9%	39.8%	**1943**	45.8%	41.5%
Northern Red Sea	**334**	41.6%	15.0%	**1544**	27.0%	7.9%	**1878**	29.6%	9.2%

**Total**	**1226**	**58.6%**	**48.3%**	**6564**	**46.4%**	**39.3%**	**7821**	**48.4%**	**40.8%**

The use of malaria prevention methods by pregnant women was high in Anseba. About 81.3% of pregnant women slept under ITNs with 76.6% having slept under an ITN during the previous night (Table 7). In Anseba, attendance of antenatal clinic was also high for the first and second visits only. In all *zobas *the use of chemoprophylaxis for malaria prevention during the present or last pregnancy was very low reaching 18.2% in Gash Barka. This is compared to the use of drugs to prevent other illnesses reported by 54.5% of all pregnant women in the same *zoba*.

**Table 7:** Malaria prevention indicators for pregnant women by *zoba*

		**Sleeping under Net/ITN**			**Receiving Antenatal care**			**Receiving specific chemoprophylaxis**		
		
								Anti-malarial		
									
**Zoba**	**N =**	Any Net	ITN	ITN in last 6 months	At least 1 visit	1–2 visits	3–6 visits	Used	Correct dose	Other illness
Anseba	64	81.3%	81.3%	76.6%	78.1%	65.6%	12.5%	3.1%	3.1%	23.4%
Debub	61	59.0%	59.0%	59.0%	73.8%	41.0%	32.8%	0.0%	0.0%	19.7%
Gash-Barka	55	60.0%	56.4%	56.4%	83.6%	63.6%	20.0%	18.2%	14.5%	54.5%
Northern Red Sea	58	29.3%	13.8%	6.9%	77.6%	58.6%	19.0%	0.0%	0.0%	31.0%

**TOTAL**	**238**	**58.0%**	**53.4%**	**50.4%**	**78.2%**	**57.1%**	**21.0%**	**5.0%**	**4.2%**	**31.5%**

During the previous six months, IRS was carried out to a small extent mostly in Gash Barka where 42.2% of the households were sprayed. This was followed by Debub (25.8%) and Northern Red Sea (3.2%) giving a national average of 18.1%.

**Figure 5:**
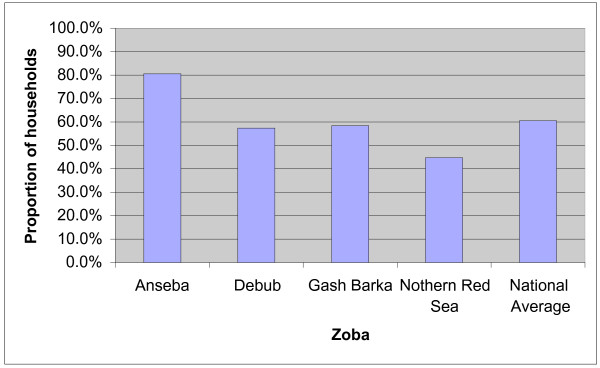
Proportion of households participating in ecological management by zoba.

Community members actively participated in malaria control activities in the endemic zobas. More than 80% of the households in Anseba participated in such activities (Figure 5).

### Findings from facility surveys

At the facilities, health workers requested laboratory tests in 81% of the patients suspected to have malaria. The diagnostic skills of laboratory staff were high as they realised blood slide examination sensitivity of 99.1% and specificity of 95% when cross-checked for quality control by the national reference laboratory.

The quality of patient care did not show significant improvement within the facilities. Data from health facility survey shows that only 52% of patients with suspected malaria had a temperature measurement taken, 75% were checked for pallor while only 58% were managed according to the national guidelines.

Although all facilities surveyed had adequate supplies of the recommended combination therapy of chloroquine and sulfadoxine-pyrimethamine (fansidar) 7% of the patients surveyed were prescribed chloroquine alone. At the same time, in 2001, only 7.2% of infected persons sought and obtained effective treatment within the recommended time of 24 hours. There was little change in this proportion, rising only to 7.5% in 2004.

### Linear regression analysis results

Results of Time series model analysis (ARIMA) (Table 8) show strong negative correlation between the numbers of ITNs distributed (β = -0.125, p <0.005), and the amount of DDT and Malathion (in kg) used in IRS (β = -2.352, p < 0.05) with the overall reduction in malaria morbidity. The effect is sustained in model fit where these two factors are included. However this joint effect is not statistically significant. The data was inadequate to compute a model to which rainfall was added to ITNs, and IRS.

**Table 8:** Time series analysis (ARIMA) of cases and deaths, against malaria control interventions

Univariate analysis		
**Effect on malaria morbidity (number of cases)**		

	β coefficient	Probability
ITNs (number distributed)	-0.125	0.005
Number of ITNs retreated	-0.016	0.02
IRS (kg of DDT & Malathion)	-2.352	0.05
Malathion (kg)	-3.270	0.05
CHAs trained (number)	-55.483	0.6
Rainfall	275.95	0.4
Population protected	-0.728	0.11

		
**Effect on case fatality (number of deaths)**		

	β coefficient	Probability
IRS (kg of DDT & Malathion)	-0.002	0.08
Health workers trained	0.226	0.03
Abate (litres)	-0.468	0.3
		
Multivariate		
Effect on malaria morbidity (number of cases)		
	β coefficient	Probability
ITNs	-0.1663	0.13
IRS	0.832	0.45
		
Case fatality		
	β coefficient	Probability
ITNs	-0.00011	0.6
IRS	0.0006	0.9

The correlation between the total number of health personnel trained and the trends in malaria morbidity and case fatality rate decline was statistically significant (p < 0.028).

There was an overall reduction in the average annual rainfall in the country during the study period. The observed association between the decline in rainfall for the period 1999–2004, and the reduction in case fatality and malaria incidence rates were not statistically significant (p < 0.441).

The negative association between case fatality and IRS, population protected, number of health workers trained and larvicidal activities was not statistically significant.

## Discussion

HMIS and cross-sectional survey data were used to assess the effects and impact of Eritrea's Roll Back Malaria Programme for the period 2000–2004. The goal of the programme was to reduce morbidity and mortality due to malaria to such low levels that malaria was no longer a public health problem in the country [12]. In five years, the programme exceeded the national targets of 80% reduction in malaria morbidity and mortality and surpassing the 60% objective of households owning ITNs. The achievements are also well above the Abuja targets for 2010.

The thrust of malaria prevention is to reduce human mosquito bites. This can be achieved through use of ITNs or reduction in either or both of the larvae and adult mosquitoes [1]. In Eritrea, the use of ITNs contributed most to the reduction in malaria morbidity and mortality. The number of ITNs distributed as a proxy for the reduction in human bites was strongly correlated to the observed steep decline in both morbidity and mortality for the period 2000–2004. Access to ITNs was rapidly increased by targeting pregnant women using the antenatal services and through freely availing ITNs to vulnerable groups of women and children below the age of five. Antenatal attendance in the country is estimated to be 70% for at least one visit during pregnancy. Free ITNs were distributed to the vulnerable groups and internally displaced populations, initially as a pilot project [18]. By 1999, free ITNs distribution had been extended to all the high-risk malaria zones and there was a sustained effort with a target of ensuring that in at least 70% of the population at risk, each household had a least two ITNs. The reported ITN coverage was in excess of 80% in most regions especially the high malaria endemic areas. In Senegal and Nigeria where the thrust is to encourage commercial supply of ITNs and creating demand among consumers and reaching vulnerable populations though targeted subsidy programmes, household coverage of ITNs was reported to be comparatively low at 43% and 10%, respectively [19].

Arguably the most cost effective tool in malaria prevention is the use of ITNs. Randomised studies have documented up to 30% reduction in the number of under-5 deaths through ITN use alone [20]. In a related report it was concluded that 6 deaths are averted for every 1,000 children age 1–59 months that sleep under ITN [4]. In Eritrea, ITNs use as a single intervention was strongly correlated to the 84% decline in morbidity and mortality. In view of the high effectiveness of this method three related issues needed to be assessed: usage of nets by children and pregnant women, re-treatment of ITNs, and sustainability.

It is also clear that the population is getting motivated to use the ITNs regularly. The proportion of children sleeping under ITNs in Eritrea is high compared to Senegal or Nigeria where only 25% and less than 5% children below the age of 5 years sleep under ITNs [18]. In other settings it has been shown that children are disadvantaged in resource allocation [21]. In Eritrea this does not appear to be the case. Although the proportion of pregnant women sleeping under ITN is only 50%, this is probably the highest in the continent.

ITNs re-treatment was equally high at 62% (done within the previous six months of the survey). This can be attributed to community involvement and token monetary incentives given to community health agents in return for increased ITN re-impregnation rates [22]. For each ITN re-treated, the CHA received 40 cents-Nakfa (3 cents US). The observed decline in the ITNs re-treatment may not affect the programme as the country is in the process of introducing Long Lasting Insecticide-Treated Nets.

One important finding of the study in respect to sustainability was that in Eritrea, 23.7% of the households surveyed in 2004 had purchased the ITNs [22]. It is plausible that the perceived beneficial effect of ITNs freely distributed in the community has positively influenced some households to invest in ITNs. Moreover, in Eritrea one free ITN is considered cost-effective given the lifetime cost of treating malarial infections and the life-saving effect of ITNs. Therefore, the government has annual running budget-line for procurement of ITNs and their free distribution to high-risk population groups.

IRS was the next most important vector control method in the country. Approximately 13% of the population in malaria risk areas of the country benefited from IRS. DDT and Malathion were the two chemicals commonly in use. Effective community mobilization and involvement contributed to the observed increasing IRS coverage. Controversies surrounding the use of DDT, which was the mainstay of eradication and vector control, have tended to undermine success in the tropics [1,23]. Recent shifts in favour of controlled indoor use of DDT have supported renewed interest leading to its re-introduction in some countries including Eritrea. The Roll Back Malaria programme in Eritrea advocates for the use of DDT for IRS alongside experimental preparations. Although the use of DDT is contestable given the environmental risks it poses, many of the poor countries cannot afford the alternative chemicals currently being tested, as their cost is prohibitive [24]. In addition tests in Eritrea on resistance to the commonly used insecticides do not show any evidence of resistance to DDT.

Although ecological management activities were statistically not associated with significant effect on either morbidity or mortality due to malaria, other studies have shown that trampled pools, rain pools, ponds, dams, swamps, drainage channels and communal water supply points are favorable larval habitats for *anopheline *mosquito in Eritrea [25]. In addition, there is strong correlation between adult mosquito density and larval density. Elimination of the known habitats was therefore an important primary preventive measure for malaria. The environmental control measures included covering of breeding sites for mosquitoes and using larvicides for water bodies that were not amenable to covering. The relatively large water collections tended to be reservoirs for the vector during the dry season. In 2004, more than 80% of the breeding sites in Southern Red Sea, Northern Red Sea and Anseba were covered through active community participation [18]. The remainder was subjected to temephos, a larvicidal chemical. In areas where it was not possible to cover the sites, use of larvicides was found to be a favorable alternative because the water bodies are relatively limited in Eritrea, especially in Gash Barka, Debub and Maekel. The role of the community was central to the success. Nearly 60% of the adult population in the four high-risk zobas surveyed, participate in environmental management activities.

Provision of early clinical diagnosis and laboratory confirmation, are other critical aspects of effective and efficient case management. The training given to laboratory technicians working in hospitals at the *zoba *referral hospitals and *sub-zoba *facilities on microscopy equips them with adequate skills. Malaria diagnostic acumen at the facility level is very high since at least 80% of the diagnoses are confirmed by laboratory tests carried out by well-trained technicians. The National Laboratory quality assurance check confirmed high sensitivity (99.1%) and specificity (95%) of these malaria tests. The thick and thin peripheral blood smear with Giemsa stain is considered the gold standard for the diagnosis of malaria, achieving overall 90% sensitivity and specificity [26].

The quality of clinical case management did not change and could not have contributed to the fall in case fatality rate. These findings are consistent with earlier surveys in Eritrea [27]. This is probably related to the high attrition rates resulting from internal transfers of cadres of staff and insufficient training on integrated management of childhood illnesses (IMCI). In addition the health seeking behaviour of the population showed little change during the study period. In 2004 only 7.5% of the sampled sick children received treatment within 24-hours of the onset of fever reflecting 6% rise from 2001 level. Access to health facilities and information are some of the contributing factors to the slow change in health seeking behaviour. The NMCP developed a malaria communication strategy following KAP survey conducted in 2002 but implementation of the strategy only commenced in 2004 [22]. The observed decline in case fatality after 2002 following an initial rise between 2000 and 2002, may be due to the change of policy on the first line treatment from chloroquine alone to a combination of chloroquine and suphadoxine-pyrimethamine.

CHAs are increasingly diagnosing and managing malaria cases. The 50% rise in their contribution to case management in Eritrea is remarkable. CHA are locally based and trained people who are easily accessible and culturally accepted in the locality [18]. This cadre became a revelation and asset of the NMCP in terms of effectiveness through early diagnosis and prescribing of relatively cheap and safe management regimens [13,18]. Training of CHAs and those at other health facilities was an ongoing exercise improving capacity and competence to manage simple cases of malaria and enable recognition of early warning signs for severe malaria for referral to better-equipped centres [13]. The CHA programme currently suffers from lack of standardization since the CHAs rely only on clinical diagnosis. The availability of reliable Rapid Diagnostic Kit, which is already in use in the country, is expected to become a useful tool in the services the CHAs provide within the community. The Rapid Diagnostic Test Kit has been found to be easy and cheap for on-site use and particularly so in countries like Brazil that have adopted aggressive active case detection [28,29].

Surveillance for emergence of resistance to first line anti-malaria drugs particularly chloroquine and first line combination drugs (chloroquine plus sulphadoxine-pyrimethamine), is important for programme success and reducing case fatality and an adjuvant to improving quality of care. Drug resistance surveillance is also essential in order to increase the useful therapeutic life of a constituent drug [30]. However, for this to be effective strong systems and decisive management are required for timely and effective response. Eritrea has established twenty centres, which in 2002 enabled the NMCP to change the first line treatment to the combination of chloroquine and sulphadoxine-pyrimethamine. Chloroquine, until recently, the mainstay of malaria treatment, precluded its use as a single drug in Eastern Africa [4]. In Eritrea, resistance to the new first line regimen is still low.

Surveillance and epidemic preparedness systems are well entrenched in Eritrea's malaria control programme as a means of reducing morbidity rates and case fatality. The NMCP collects rainfall data daily, continuously analyses it and the results are fed into the national and the *zoba *coordinating office databases. During the decade, the initial decline in the amount of the annual rainfall in the country stabilised at between 300 and 400 mm. Although the extent to which this influenced malaria morbidity and mortality trends was not statistically significant, this finding is inconclusive, as the study period coincided with a persistently lower than normal rainfall averages for Eritrea. In many countries rainfall and temperature data are predictors of impending epidemic and serve as early warning system. In East Africa, climate variability has been shown to contribute to the likelihood of an epidemic [31]. The precision of rainfall and temperature data and lead-time can be enhanced by satellite based meteorological data [32]. Eritrea is not accessing satellite data as a routine source but uses data from weather stations spread all over the country.

For epidemic preparedness, Eritrea monitors the number of new malaria cases during each month. An impending epidemic is suspected once the reported number of cases per month in a health facility first rises higher than the third quartile of the number estimated for that facility. The last epidemic reported in the country was 1998 [9]. The absence of epidemics in the intervening period cannot be explained on the basis of climatic factors alone. In the case of Eritrea the resultant control of epidemics using the ecological management and household protection may be the mechanism through which epidemics were prevented. The sensitivity of the epidemic thresholds has been questioned leading to the proposed replacement of the quartile measure by the use of weekly averages. The occurrence of severe epidemics is to be expected with the dramatic decline in the malaria incidence in the country and consequent to the declining immunity in the population. Severe *Plasmodium falciparum *malaria is known to occur in low transmission intensity [33]. Seasonal and unstable malaria transmission, attend to similar phenomena [34].

The final question to be explored was the role of each of the interventional measures in reducing morbidity or mortality. Within the limitations of the current study design it is evident that combining ITN use with IRS or other vector control measures did not confer added value to the outcome in malaria mortality or morbidity. This is not surprising since *An. arabiensis *is endophilic, and both methods act at the point of breaking the vector-human contact. This is supported by observations from elsewhere that DDT spray can eliminate up to 93–95% indoor resting density of a vulnerable vector [23].

In summary therefore, the Abuja Declaration targets for the Roll Back Malaria initiative were met on schedule because the government had set even higher targets for itself. There was extensive community and personal prevention measures which started as donations of ITNs, initially targeted at pregnant women and children and sustainable through community awareness. Both morbidity and mortality have declined to a point where malaria ceases to be a major infectious disease in this small African country. This is remarkable as no other country in the continent has similar achievements.
